# BEING A CAREGIVER DOES NOT INCREASE MORTALITY RISK AT 10-YEAR FOLLOW-UP IN A NATIONAL US SAMPLE

**DOI:** 10.1093/geroni/igad104.3201

**Published:** 2023-12-21

**Authors:** Dominique Cheung, Atina Manvelian, Erin Bouldin, Joseph Wielgosz, Ranak Trivedi

**Affiliations:** VA Palo Alto Health Care System, Palo Alto, California, United States; Santa Clara University, Santa Clara University, California, United States; University of Utah, Salt Lake City, Utah, United States; VA Palo Alto Health Care System, Palo Alto, California, United States; Stanford University, Stanford, California, United States

## Abstract

An increasing number of people are expected to become caregivers of relatives with medical or mental health conditions. The chronic stress of family caregiving has been shown to worsen one’s health, leading to concerns that it may also increase mortality risk. Seminal work showed associations between caregiving and increased mortality risk, but recent population-based studies have disputed this. Our objective was to use longitudinal, nationally representative data to rigorously examine whether caregiver status was associated with 10-year mortality. We used data from the National Social Life Health and Aging Project (NSHAP), a nationally representative study of 2,367 older adults (57-85 y) that combines interviews, surveys, and mortality data. We compared 10-year mortality risk between self-reported caregivers and non-caregivers with and without propensity score matching. Baseline sample included 401 caregivers (68.6±7.6 y) and 1,966 non-caregivers (69.2±7.7 y). Among caregivers, 25.0% had 2+ chronic health conditions, compared to 41.1% of non-caregivers. At the 10-year follow-up, 20.6% of caregivers had died versus 29.6% of non-caregivers (p<.0001). After propensity score matching on demographics, health, and limitations in? activities of daily living, there were no differences in mortality between the groups (p>0.05). Our study suggests no difference in mortality risk based on caregiver status. While family caregivers frequently experience negative impacts on well-being because of their role, it is heartening to know that caregiving may not confer an additional risk of death.

